# Biological Use of Nanostructured Silica-Based Materials Functionalized with Metallodrugs: The Spanish Perspective

**DOI:** 10.3390/ijms24032332

**Published:** 2023-01-25

**Authors:** Diana Díaz-García, Sanjiv Prashar, Santiago Gómez-Ruiz

**Affiliations:** COMET-NANO Group, Departamento de Biología y Geología, Física y Química Inorgánica, ESCET, Universidad Rey Juan Carlos, C/Tulipán s/n, E-28933 Móstoles, Spain

**Keywords:** metal complexes, nanostructured materials, silica, therapy, cancer, antibacterial, drug delivery

## Abstract

Since the pioneering work of Vallet-Regí’s group on the design and synthesis of mesoporous silica-based materials with therapeutic applications, during the last 15 years, the potential use of mesoporous silica nanostructured materials as drug delivery vehicles has been extensively explored. The versatility of these materials allows the design of a wide variety of platforms that can incorporate numerous agents of interest (fluorophores, proteins, drugs, etc.) in a single scaffold. However, the use of these systems loaded with metallodrugs as cytotoxic agents against different diseases and with distinct therapeutic targets has been studied to a much lesser extent. This review will focus on the work carried out in this field, highlighting both the pioneering and recent contributions of Spanish groups that have synthesized a wide variety of systems based on titanium, tin, ruthenium, copper and silver complexes supported onto nanostructured silica. In addition, this article will also discuss the importance of the structural features of the systems for evaluating and modulating their therapeutic properties. Finally, the most interesting results obtained in the study of the potential therapeutic application of these metallodrug-functionalized silica-based materials against cancer and bacteria will be described, paying special attention to preclinical trials in vivo.

## 1. Introduction

The continuous scientific evolution in the search for simpler and improved solutions in different fields of knowledge has meant that nanotechnology has played a very important role in recent decades. Already in 1959, the American physicist Richard Feynman mentioned in his speech “There’s Plenty of Room at the Bottom”, about the enormous possibilities of the use of unexplored nanotechnology and nanoscience. The term nanotechnology was assigned to the Japanese professor Norio Taniguchi in 1974 and refers to the design and/or manipulation of matter in the nanometer range (1 × 10^−9^ m).

The development of nanoscale materials (nanomaterials) has had a major impact on the fields of science and engineering in general, influencing new technologies and providing industrial and medical solutions. Nanomaterials can be of natural origin (proteins, some carbon materials, etc.) or artificial (metal nanoparticles, oxides, etc.), which are designed to have specific functions, such as increasing resistance, chemical reactivity, conductivity or improving biomedical treatments by acting as drug carriers.

In the field of nanomaterials for biological applications, liposomes are the most utilized nanostructured vehicles due to their double lipidic layer which permits the transport of diverse molecules of interest enclosed inside, coupled outside or even between both layers [1]. In fact, these types of nanoparticles were the first nanodrug approved by the FDA in 1995 under the name of Doxil^®^, for the treatment of Kaposi’s sarcoma, ovarian and breast cancer [2]. Since then, there have been dozens of combinations of liposomes and lipid nanoparticles, PEGylated and non-PEGylated, with an approved application for biological purposes [3]. For cancer therapy, the most recently FDA-approved nanodrugs were, in 2015, a PEGylated liposomal system with irinotecan, namely, Onivyde^®^, for the treatment of metastatic pancreatic cancer [4], in 2017, Vyxeos^®^, a formulation of cytarabine and daunorubicin for myeloid leukaemia application [5] and, most recently, in 2018, Onpattro^®^, the first combination of RNAi therapeutic delivery encapsulated in a lipid nanoparticle [6]. In this context, albumin particles also have some examples of approved FDA nanosystems such as Abraxane^®^, a delivery system of paclitaxel [7].

Currently, there are many inorganic materials that are the object of study for biological applications and specifically as drug-containing systems. For example, there are magnetic nanoparticles, approved for the treatment of brain tumors or also used as a contrast agent in nuclear magnetic resonance studies or iron-based colloids for the treatment of iron deficient anaemia [3]. Other metal nanoparticles have also been extensively studied in medicine, but they are still in the first stage of clinical trials. For anticancer purposes, gold [8] and platinum nanoparticles [9,10] are the most common in the literature, although there are not so many metal systems used against cancer with theragnostic applications [11]. Copper and silver nanoparticles are the most effective for antibacterial activity [12]. For bone regeneration and treatment applications, there is not a specific metal used but copper, silver, platinum or even cobalt have been studied [13,14,15].

Due to their versatility, one of the paramount inorganic materials are nanostructured silicas, which have a wide range of applicability from the manufacture of nanodevices, catalysis and photocatalysis to various biomedical applications. In this review, we will focus on various applications in the field of medicine, such as cancer, antibacterial activity and other biological studies.

Silica materials, in particular mesoporous silicas, have attracted the attention of the entire scientific community due to their exceptional physicochemical properties such as mechanical strength, chemical stability, biocompatibility and synthetic versatility. These nanomaterials are made up of a SiO_2_ matrix and are characterized by the presence of pores with a diameter between 2 and 50 nm. These unique characteristics provide silicas with two distinct domains: an outer and an inner surface in the pores, which offers multiple functionalization options. These exceptional properties led the Spanish group of María Vallet-Regí to develop the first drug delivery system based on a mesoporous silica (MCM-41) loaded with the anti-inflammatory drug ibuprofen [16]. This pioneering work opened a new field of biomedical applications for this type of inorganic material.

## 2. Metallodrugs as Biological Agents

The enunciation of coordination theory by Alfred Werner (Nobel Prize 1913) opened doors to new fields of research in inorganic chemistry. Peter Sadler’s research group, which started studying the role of metals in medicine through the design and chemical mechanism of action of therapeutic metal complexes, is considered one of the fathers of metals in medicine [17] together with Prof. Rosenberg, who discovered cisplatin anticancer behavior [18].

Medicinal organometallic chemistry is the discipline dealing with the interaction between metal-based therapeutics and biomolecular targets. The application of inorganic chemistry to medicine can provide complementary aspects to the biochemical and physiological mechanisms already known from organic chemistry, due to the specific properties offered by metal ions and their ability to interact and covalently bond to biomolecules.

The first metal complex applied in the medical field was cisplatin. Since then, numerous platinum complexes as well as other metal derivatives (Ru, Au, Ti, Sn, etc.) with various applications in this field have been added.

Table 1 shows some representative biological applications to which Spanish authors have contributed by designing complexes with different metal nuclei, as a potential improvement or alternative to conventional chemotherapeutic treatments.

Metal complexes have been and continue to be an attractive alternative to conventional therapies due to the possibility of modifying the metal, oxidation state, coordination number and ligands. The mode of action of these complexes is quite broad, ranging from directly attacking DNA (metalation), inducing cell death by apoptosis, increased generation of reactive oxygen species (ROS) or by activation and/or inhibition of enzymes and genes [27]. However, undesirable side effects, including nephrotoxicity, neurotoxicity and gastric disorders, strongly limit the dosage.

These important drawbacks, together with the low solubility of most metal complexes, make it imperative to find an alternative strategy for the administration of metallodrugs. A promising approach is encapsulation, or covalent functionalization, in biocompatible materials, designing controlled-release systems. Silica-based materials are the most promising inorganic systems as they have a high loading capacity, a much higher capacity for protection against degradation of the encapsulated substance than traditional systems and the targeted release that can be achieved upon a given stimulus [28].

## 3. Application of Metallodrugs in Silica-Based Materials

Silica materials are characterized by mesoporous cavities that accommodate and protect a wide variety of molecules, while on the surface they can be further functionalized with agents that improve their biodistribution, dispersibility, selectivity and specificity. Therapeutic agents can be adsorbed and/or covalently linked to both the interior walls of the mesopores or to the external surface of the particles. For this reason, mesoporous silicas are considered excellent candidates for clinical applications such as multifunctional nanoplatforms which may help in the design of nanodevices capable of performing therapy and diagnosis simultaneously (theranosis). Today, research into the synthesis, optimization and functionalization of silica nanoparticles is a very active field that has reported numerous ways of synthetic methods for the preparation of silica of different morphology, size or porosity. The control of the structural features of the nanostructured silica is, of course, crucial for the final application, and a rational use of the synthetic method is required for obtaining interesting systems for biological purposes. In this context, the most common procedures for obtaining mesoporous silica are the sol-gel methods used, e.g., for the synthesis of SBA-15, MSU-2, KIT-6, HMS and MSN silica systems. In addition, some other alternative synthesis routes, such as hydrothermal or solvethermal synthesis (for MCM-41, MCM-48 and FSP), ultrasound-assisted synthesis or procedures using microwaves, have also been employed and have been summarized in a recent review [29].

Thus, apart from the research on the optimization of novel synthetic procedures for obtaining silica with made-to-measure structural features, in the last fifteen years, systems based on mesoporous silica nanoparticles (MSN) have been studied as transport vehicles for metallodrugs, in order to avoid the disadvantages associated with the direct use of metal complexes in therapy. Due to the biocompatibility and nontoxicity of the starting silica, the therapeutic activity in these materials is provided, in general, by the metallic load of the system, either in the form of a metal complex or a metal nanoparticle. Therefore, the results of biological studies determining the cytotoxic activity in silica-based materials is expressed in terms of the IC_50_ of the whole material and as a function of the metal load of each system, so that the systems can be compared by the metal IC_50_ concentration.

There are a fair number of reviews describing the different methods of synthesis of nanostructured materials [30,31] and their application as drug or gene delivery systems [32,33,34] or in other biomedical applications [28,35,36]. However, in this context, only the highly cited review of our research group, published in 2016, describing the state of the art of nanostructured materials (liposomes, ceramics and carbon nanomaterials, etc.) as anticancer agents with metallodrugs mainly in cancer therapy has been focused on the combination of metallodrugs and nanosystems against cancer [37]. Thus, in the last few years, and to the best of our knowledge, the literature lacks a specific review revisiting the studies carried out during the last 5–6 years in the field of nanostructured silica materials functionalized with metallodrugs and their use in anticancer and antibacterial applications.

Therefore, in this review, our efforts are focused on summarizing the latest advances in metal- and metal complex-functionalized nanostructured porous silica, as well as in the silica systems encapsulating metallodrugs, presenting their anticancer and antibacterial potential, with special regards to the contribution of Spanish groups in this exciting field of research for the scientific community.

### 3.1. Anticancer Therapy

Cancer is defined as the process of uncontrolled growth and spread of cells causing an abnormal increase in the mass of the affected tissue, commonly known as a tumor. These cells have a great capacity for proliferation, being able to invade other tissues (metastasis). In addition, cancer cells can lose differentiation being, therefore, capable of surviving in atypical environments. According to the data of the World Health Organization (WHO), cancer is the second highest cause of death worldwide, with 18.1 million incident cases in 2018 and with an estimated growth of new cases of more than 63% in 2040. Even in a pandemic context, as in 2020, cancer (602,350 deaths) was still the second highest cause of death in the United States behind cardiovascular diseases (696,962 deaths) and ahead of COVID-19 (350,831) [38].

**Figure 1:**
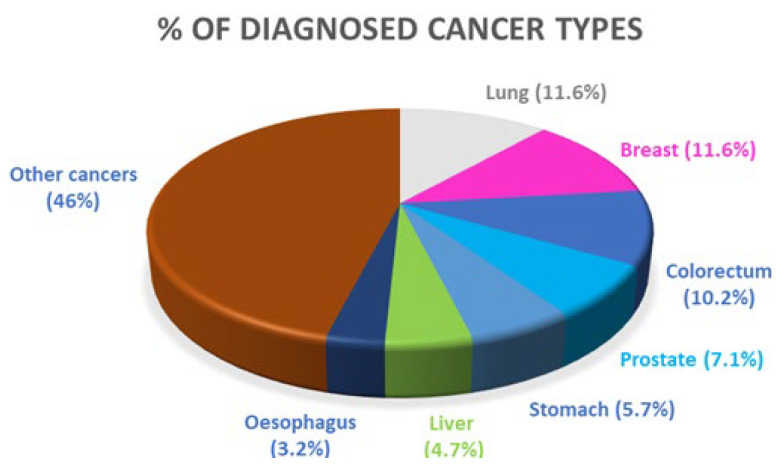
Percentage of each diagnosed cancer type. Data from the International Agency for Research Cancer, World Health Organization.

Cancer can start to develop almost anywhere in the body, but the most diagnosed cancer types are lung, breast and colorectal (Figure 1).

There are different treatments with the choice depending on the type of cancer and the progression of the tumor. Chemotherapy is the most widely used treatment and is based on the use of drugs to destroy and stop cancer. Some of the most used drugs for chemotherapy treatment are cyclofosfamide (Cytoxan^®^), daunorubicin (Cerubidine^®^, Daunoxome^®^), doxorubicin (Adriamycin^®^, Doxil^®^) or paclitaxel (Taxol^®^). However, since the fortuitous discovery of the cellular inhibition capacity of the compound cisplatin by Rosenberg in 1964 [18], the door was opened for the study of metal complexes against cancer. Cisplatin, commercially called Platinol^®^, was the first metal complex used to treat different types of sarcomas and carcinomas. The search for platinum compounds that had cytotoxic activity was expanded, leading to other systems that have been approved and are commercially available such as carboplatin (Paraplatin^®^) or oxaliplatin (Eloxatin^®^).

However, the use and study of metallodrugs in the field of cancer therapy has not stopped here. There are many alternative metals which have anticancer therapy potential such as titanium, tin, ruthenium, gold and gallium, among others [39].

#### 3.1.1. Platinum Compounds

**Figure 2:**
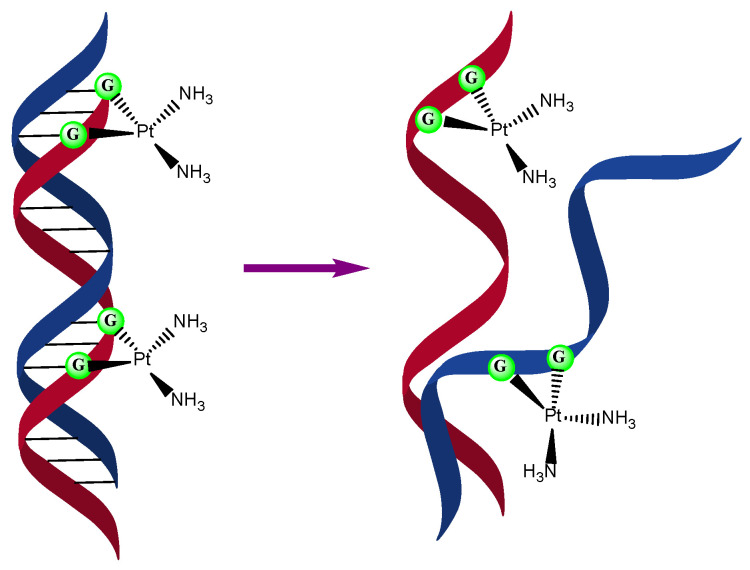
Interaction of the compound cisplatin with DNA through guanines (**left**) and its denaturalization (**right**).

The mechanism of action of cisplatin is one of the most studied in the field of metallodrugs as it is the first metal compound used in chemotherapy; even so, the cellular uptake mechanism and the apoptosis induction in cancer cells are still not clear. Some studies reported that cisplatin enters inside cells by passive diffusion and others show evidence of cisplatin uptake due to protein interactions (copper-transporting proteins), to enter inside the cells. It is well known that the primary target of platinum compounds is DNA, forming Pt-DNA adducts and, as a result of that, generating DNA damage and G2 arrest. Pt forms normally, covalent bonds with the N^7^ of two guanines which have a better thermodynamic stability than adenines (Figure 2). This DNA distortion generates changes in the high-mobility groups (HMG) in charge of the protein repair transcription factors and other DNA pathways. Specifically, HMGB1 is directly connected with the p53 protein (tumor suppressor protein). On the other hand, cisplatin can induce the accumulation of other apoptosis suppressors, the p73 protein and the activation of p38, which has an important role in the response to stress stimuli. However, although DNA is the major target of Pt-based compounds, only less than 10% is able to reach this macromolecule.

Platinum compounds have high affinity for sulfur atoms, therefore interacting with biomolecules such as cysteine and methionine, and generating a strong Pt–S interaction. This process causes competing reactions and the amount of drug that reaches the target area is, therefore, very low, also causing Pt–protein interactions that may damage the kidney. Although a Pt release sometimes occurs that interacts with the sulfurs to the DNA guanines, the control of the activity in the S-donor molecules is very important. In the treatment with Pt-based drugs, protective agents containing S-donors are used in order to decrease the secondary reactions. Some cisplatin analogues have shown some differences in their activity and mechanism of action, showing some advantages. Oxaliplatin, for example, presents a good anticancer activity although it has less cytotoxicity because oxaliplatin is not recognized by some repair proteins of DNA and it generates immunogenic cell death [40]. However, carboplatin requires higher doses than cisplatin and generates resistance in spite of it being less toxic than oxaliplatin. Thus, there are several binding parameters and interaction behavioral aspects that make this kind of compound still very intriguing.

Despite the expectations generated by cisplatin analogues, Pt compounds present some drawbacks, such as side effects (nephrotoxicity and neurotoxicity), as well as the resistance generated after their successive administrations limiting their application. As a result of that, clinical treatment with platinum compounds is usually administered as a combination of several drugs or in combined therapies. For example, very recently, the FDA approved in the USA the use of a novel drug, namely, lurbinectedin (Zepzelca^®^), for the treatment of metastatic microcytic lung cancer after a Pt-based treatment [41]. The use of nanovectors as vehicles of Pt compounds transport improves these disadvantages. In this context, there are a lot of vehicles used in the transport of cisplatin and its derivates such as polymeric micelles or dendrimers [32], but the inorganic nanoparticles are gaining importance due to the extensive work that is being carried out to understand their behavior in biological systems. Specifically, mesoporous silica nanoparticles have been considered a very promising option for the encapsulation and subsequent delivery of cisplatin and its analogues. MCM-41 mesoporous silica was the first silica-based nanoparticle used in the adsorption of an FDA-approved Pt compound (carboplatin) in aqueous suspensions [42]. The system showed that the amount of the platinum drug incorporated into the MCM-41 was only 1.8%, a low quantity compared to other drugs loaded in the same silica. This study exhibited the importance of the solvent in the adsorption process because the use of less-polar solvents seems to allow more drug adsorption on silica. Subsequently, Z. Thao et al. went further and studied different systems based on MCM-41 and SBA-15 loaded with cisplatin or its *trans* isomer, transplatin. These materials showed different adsorption behavior and therapeutic effects against leukaemia, obtaining better results in cell viability than Pt compounds and doxorubicin alone after 24 h of incubation [43]. In addition, T. López et al. loaded a cisplatin derivative on small mesoporous silica nanoparticles for studying the catalytic degradation of DNA, observing a tumor suppression attributed to high DNA damage caused by this type of system [44].

Another approach to transport Pt derivates into silica materials is an immobilization of a Pt prodrug in the silica via surface modification. For example, the incorporation of carboxylic groups on the surface allows to create a pH-responsive material that, in acidic conditions or in high concentrations of Cl^-^, produces a Pt-compound release, conjugated through the ligand with carboxyl groups [45,46]. The use of these silica systems allows a combination of different drugs in the same platform in order to analyze their synergistic effects, to improve the cytotoxicity and try to reduce the dose of each drug to a minimum. H. Li and co-workers studied the combination of cisplatin and doxorubicin in modified MSN with a pH-responsive shell for co-delivery systems under an acidic environment [47], and W. Zhang et al. studied the conjugation of cisplatin and a photosensitizer (chlorin e6) for the combination of chemotherapy and phototherapy at the same time. The cytotoxic activity in these silica platforms was 47 times more active than cisplatin and, after a light irradiation, the reactive oxygen species (ROS) increased exponentially [48].

**Figure 3:**
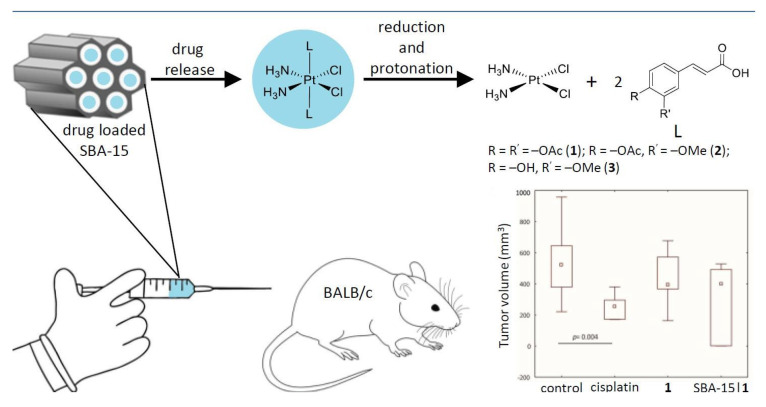
Vehiculation of platinum(IV) derivatives in SBA−15 showing strong antitumor activity in mice with breast cancer [50]. Representation of the adsorption and release of platinum compounds and their effect of tumors’ growth suppression in a mouse model. Copyright © Authors of the publication (open access).

Some recent studies in the field of platinum(IV) compounds were focused on the conjugation with SBA-15. The systems showed a high antiproliferative and cytotoxic capacity against different breast cancer lines, with values between 240 and 100 times more active than the free platinum complexes [49,50]. Thanks to their enhanced permeability and retention (EPR) capacity, the systems can accumulate in the tumor zone, producing autophagy and an increasing generated reactive oxygen species (ROS) and nitric oxide (NO), demonstrating great efficacy against breast cancer, even in mouse models (Figure 3) [50].

**Figure 4:**
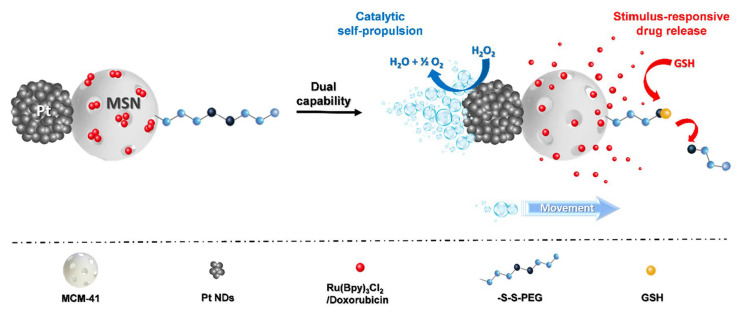
Scheme of Janus Pt-MSNs nanomotors with a catalytic self-propulsion for drug delivery by the catalytic reduction of H_2_O_2_ mediated by glutathione [52]. Copyright © Authors of the publication (open access).

The main Spanish contribution in the field of platinum derivatives loaded onto silica-based materials has been carried out, mainly, by I. del Hierro and co-workers [51] and P. Díez and co-workers [52]. The first work was supported on the covalent functionalization of a triazole ligand in a silica system that, subsequently, incorporated cisplatin to study its interaction with guanosine and albumin through electrochemical techniques, providing valuable information on the hydrolysis processes of the complex as well as analyzing the release profile in simulated biological media, observing a high stability of the silica-Pt materials. On the other hand, the work of P. Díez et al. was based on the design of nanomotors based on Janus-type silica functionalized materials with a polymer sensitive to RedOx reactions as a gate for doxorubicin release together with platinum nanodendrites with a “propulsion” capability for efficient load movement and transport and release (Figure 4).

Despite the protection that silica materials offer to platinum compounds, they still have drawbacks and the investigation in this field is still very active with the general aim of trying to reduce them.

#### 3.1.2. Titanium Compounds

A few years after the discovery of cisplatin, titanium(IV) compounds were also found to possess antitumor capacity with fewer side effects. Specifically, the studies of Köpf and Köpf-Maier showed that titanocene derivatives have an anticancer activity against different cell lines, inhibiting tumor growth considerably [53]. Due to the structure similarity with cisplatin, titanocene dichloride, TiCp_2_Cl_2_ (Cp = η^5^ − C_5_H_5_), is one of the most widely used titanium compounds tested in cancer studies together with bis[(p-methoxybenzyl)cyclopentadienyl]titanium(IV) dichloride (Titanocene Y) [54]. However, although these two systems are the most studied, there are many titanocene compounds with a variety of substituents which have shown a remarkable antitumor activity [55].

**Figure 5:**
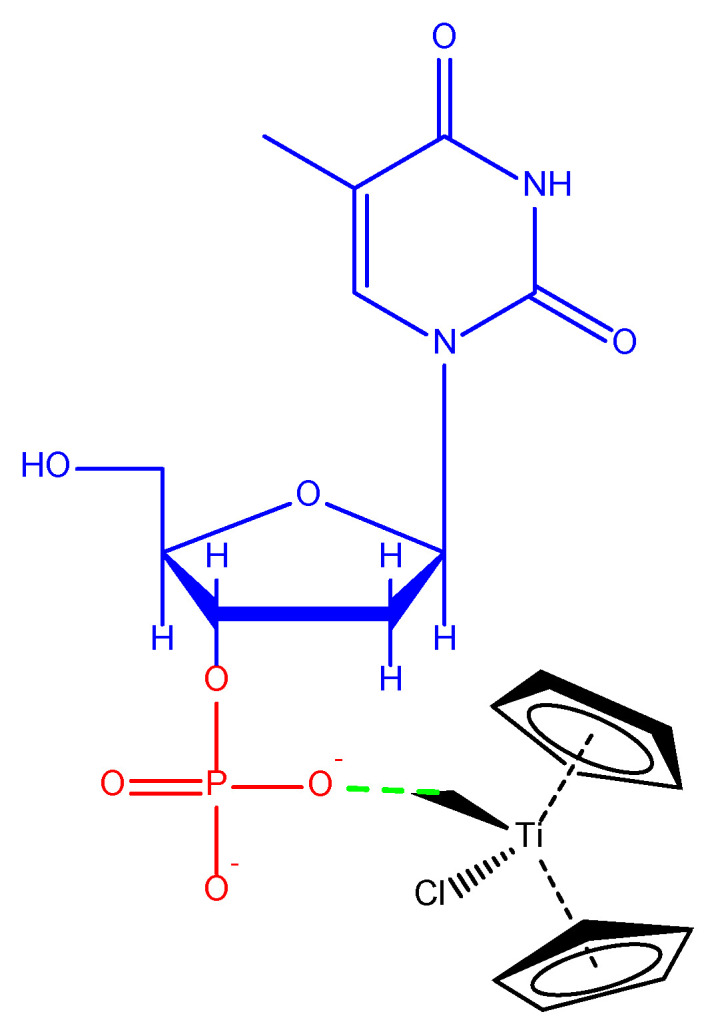
Interaction of the compound TiCp_2_Cl_2_ with DNA phosphate groups. Blue: nucleoside, red: phosphate, black: titanocene.

Even though DNA seems to be the major target, the mechanism of action is different than in the case of platinum complexes as titanocene(IV) compounds have the capacity to form DNA adducts, inducing cell cycle arrest, principally in the late S/G2 phase (although in the ovarian cancer A2780 cell line, titanocene dichloride is able to induce apoptosis in all phases). The study of the formation of Ti-DNA adducts was investigated by high-resolution mass spectrometry, finding that the preferred target for titanium(IV) compounds appears to be the oxygen of the phosphate groups of the oligonucleotides, with special interest being dedicated to thymidine (Figure 5). In all cases, the interaction with the nucleotides is pH-dependent [56].

In this context, although the mechanism of action of titanocene derivatives is still very intriguing and still developing, significant advances in understanding the role of both the ligands and the titanium center in the anticancer action via some important stu −ies involving titanocene derivatives are worthy of mention. Thus, Koubkova and co-workers evaluated the cytotoxic activity in three titanocene difluorides, observing that the mechanism of cell death induction was via autophagy and the endoplasmic reticulum stress pathway [57]. In addition, it is also well known that Ti(IV) compounds have a strong affinity for transferrin, serving this protein as a Ti transport to the interior of the cell [58,59], avoiding the appearance of insoluble TiO_2_, which decreases their cytotoxic nature [60]. Titanocene complexes also seem to be very effective against antiapoptotic genes, such as the bcl-2 and p53 family, preventing cell proliferation and having excellent antiproliferative effects in platinum-resistant cell lines [61].

In many cases, the cytotoxicity of titanium compounds is complemented by the action of another organometallic cytotoxic system or another drug of interest, with the aim of determining if potential synergistic actions between the different drugs are possible. Thus, bimetallic compounds containing titanocene and gold compounds such as Auranofin are the most common combinations [62,63]. Titanium(IV) complexes have also been studied with other cancer drugs such as chlorambucil-like drugs in order to improve the activity against different cancer cells [64].

Although the first Ti(IV) compound investigated in a clinical trial, budotitane, did not pass phase I due to the nephrotoxicity generated [65], a wide variety of titanocene complexes have been investigated in preclinical and clinical trials with promising results because of the lack of myelosuppression [66]. Despite this, after some phase II studies with some optimal titanocene-based systems, these compounds have not been developed further due, mainly, to their low solubility and their limited cytotoxic activity compared to platinum derivatives. The encapsulation of this class of compounds can improve the cytotoxic potency if systems are designed to stabilize the cytotoxic center during the transport to cells.

Most of the studies dealing with the functionalization of titanocene complexes onto silica-based mesoporous materials has been carried out by Spanish researchers, in collaboration with other groups. The pioneering work in this field was published by our group [67] and was focused on the preparation of two simple titanocene complexes, which were grafted into the silica materials MCM-41 (prepared with an hydrothermal crystallization) and SBA-15 (sol-gel method). The activity in these materials was evaluated in terms of Q_50_ (numbers of particles to inhibit the cell growth by 50%) and M_50_ (quantity of material to inhibit the cell growth by 50%). We also studied the influence that the quantity of Ti in the silica material has on the cytotoxic activity in the systems. The results showed that, with the grafting reactions, the quantity of Ti that can be incorporated is around 2% and, when raising the quantity of titanium, the cytotoxic activity in the systems against cancer cells increases [68]. In addition, subsequent work of our team evaluated the influence of different starting materials such as hydroxyapatite, alumina and two silica materials MSU-2 and HMS on the incorporation of titanocene derivates and their influence on the cytotoxic activity against a wide variety of cancer cell lines, showing that particle size has a crucial influence on the anticancer potential of the silica-based materials [69].

More recent studies of our team, in collaboration with Fischer-Fodor’s research group, have shown that a series of twenty titanocene complexes more active than the Titanocene-Y were more cytotoxic against cancer cells when they were functionalized in a nanostructured silica KIT-6. The study showed that, in all the synthesized materials, the percentage of Ti functionalized was low, maybe due to the weak acidity of the surface OH groups, although the biological study showed that the internalization of titanium inside the cancer cell is up to 45 times higher when using the KIT-6 support than when using the free metallocene complex [70].

**Figure 6:**
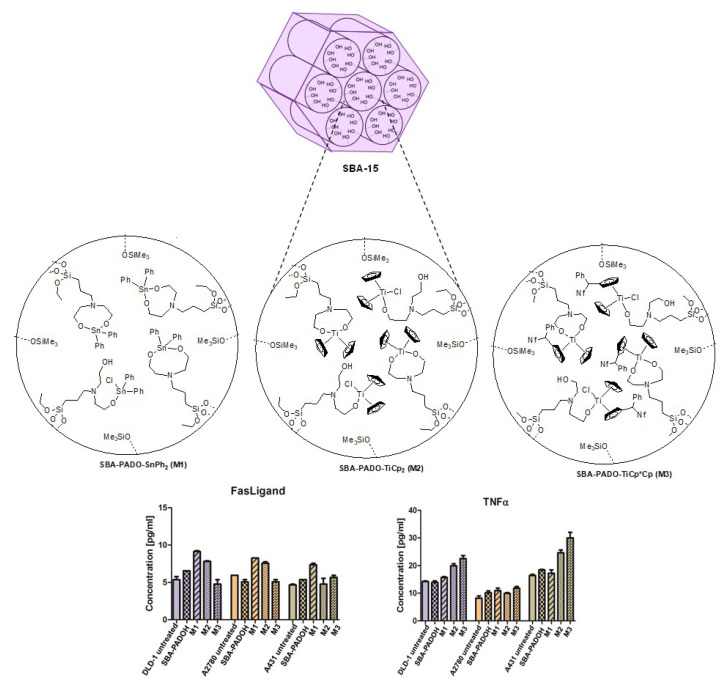
Synthesis of tin- and titanium-functionalized SBA-15 materials and mechanism of programmed cell death. Adapted from reference [73]. Reproduced with permission. Copyright © Royal Society of Chemistry.

Alternatives for the functionalization of silica-based materials with Ti complexes were used, namely, the incorporation of S-containing or OH-containing linkers grafted into silica materials. Thus, some studies of our team showed that the presence of 3-mercaptopropyltriethoxisilane grafted in MCM-41 permitted a higher percentage of functionalized Ti, further showing better antitumor activities in DNA-binding studies and in cell growth inhibition by TNFR1 modulation, than silica materials with the titanocene directly bound to the silica through the Si-OH groups (Si-O-Ti bonds) [71,72]. This strategy was also carried out using the linker 3-[bis(2-hydroxyethyl)amino]propyltriethoxysilane with TiCp_2_Cl_2_ and [Ti(η^5^-C_5_H_5_)(η^5^-C_5_H_4_CHPhNf)Cl_2_] (Nf = 2-naphthyl), observing a slow release of the metallodrugs and a trigger for apoptosis modulated by the TNF-α factor [73] (Figure 6). 

**Figure 7:**
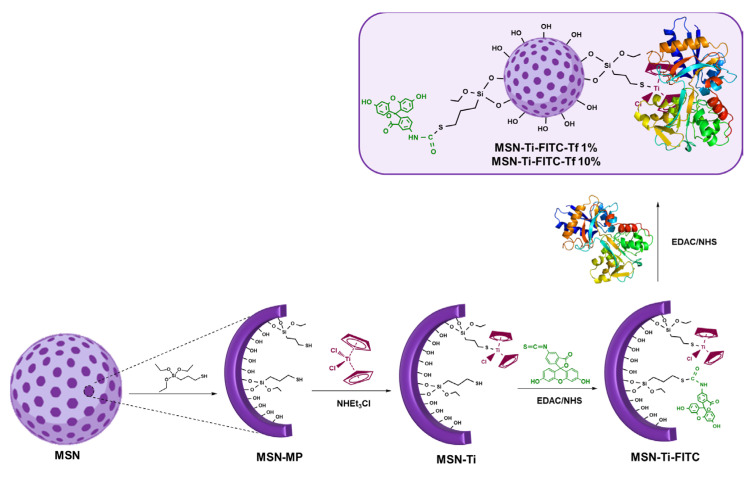
Preparation scheme of nanomaterials (purple sphere) functionalized with titanocene (organometallic compound in red), FITC (green molecule) and transferrin (multicolor macromolecule) [74]. Copyright © Authors of the publication (open access).

Recently, a study of our team [74], showed that the covalent incorporation of TiCp_2_Cl_2_ through an organic ligand with a thiol group (Figure 7) leads to an outstanding cytotoxic activity against the ovarian cancer line A2780, with IC_50_ values between 0.5–0.8 μg/mL, being, to date, the best results published for titanium-based compounds supported on nanoparticles. An extensive characterization of this nanosystem showed that the release of the metal complex is not required for such a high therapeutic activity.

#### 3.1.3. Tin Compounds

The investigation into the antiproliferative activity in other metal complexes as alternatives to platinum compounds, conducted in 1980, reported the antitumor activity in a series of diorganotin(IV) complexes [75]. Tin compounds, with a general formula R_n_SnX_4−n_, have shown the ability to induce apoptosis in a multitude of cancer cell lines. Organotin compounds are a good option as a substitute for cisplatin because they have many advantages, such as a better selectivity and water solubility, less toxicity and less adverse effects in low doses compared with platinum metallodrugs. In addition, the major improvement in organotin compounds in therapy is that cancer cells do not generate resistance against this type of system, unlike platinum compounds. This is because organotin(IV) compounds do not seem to be substrates of P-glycoprotein and, due to this, do not generate multidrug resistance (MDR) [20]. Organotin(IV) moieties have an affinity for binding with the phosphodiester backbone of DNA, thus causing damage to cells through the p53 apoptosis pathway [76]. Nevertheless, there are also studies that show that organotin compounds bind to the thiol groups of proteins, which help in improving their transport, biodistribution and enhance the antiproliferative effect [77]. Despite this, the biological activity in organotin metallodrugs is highly dependent on the type and number of ligands binding to the tin center, observing a decrease in the bioactivity as follows: R_3_SnX > R_2_SnX_2_ > RSnX_3_, highlighting with a better antitumor activity, the carboxylate derivatives and triphenyltin(IV) compounds, due to their better lipid solubility and less toxicity [20,78,79,80].

In this context, M. Vafaee and co-workers synthesized, in 2012, the first silica-based systems for the release of a nonsoluble tin(IV) coordination complex [81]. The study was carried out using MCM-41, MCM-48 and SBA-15 as supporting systems, which were modified with pyridine. The materials showed different behavior in the tin release depending on the pH, showing that at an acidic pH (similar to the stomach’s pH), completed the release of the metallodrug. Since then, other organotin complexes loaded in silicas with different morphologies and porosity have been studied. Thus, the groups of Kaluđerović and Gómez-Ruiz used SBA-15 with Ph_3_Sn(CH_2_)_3_OH, exhibiting that the silica system loaded with the tin compound needed a concentration more than three times lower to cause the same therapeutic effect compared with that of the tin compound [82]. Subsequently, Kaluđerović’s group continued studying further this system against the A375 cell line by analyzing the internalization of nanoparticles in the cells, which seems to take place mainly through macropinocytosis, and also observing the loss of the stem characteristics of the tumor cells [83]. A similar material but using SBA-15 functionalized with (3-aminopropyl)triethoxysilane as a linking system was evaluated and compared with the material in which the organotin compound was loaded by reaction with CPTS or by physical adsorption. It was observed that the functionalization with the SBA-15 is crucial in the biological activity, seeing that both systems induced apoptosis in the B16 cell line but also autophagy in the case of the amino linker and cellular differentiation in the case of the CPTS linker [84]. A more specific study was carried out using SBA-15 functionalized with the ligand CPTS and binding the organotin(IV) compound Ph_3_Sn(CH_2_)_6_OH, showing an in vivo induction of cell differentiation and a tumor growth suppression through triggering JNK-independent apoptosis in mice with B16 melanoma tumor [85].

The Spanish contribution in this kind of system is not only limited to the collaboration of our group with the group of Prof. Kaluđerović but also to the development of other systems with interesting cytotoxic properties which have been explored in vitro and in vivo with attractive results. For example, one of the first studies was focused on an SBA-15 material that was loaded first with 3-[bis(2-hydroxyethyl)amino]propyltriethoxysilane and subsequently reacted with SnPh_2_Cl_2_ in the presence of a base [73], observing the different apoptosis pathway in comparison with the titanium complexes functionalized in the same silica system (Figure 7). Even though the tin(IV) complex was bound through an aminodiol ligand, a non-negligible release (around 10%) was found in the simulated body fluid. In addition, our research team recently studied the effect of the carbon chain on covalently functionalized tin complexes in SBA-15 [86]. In vitro studies against A375 cells and B16 melanoma cells determined that all functionalized novel systems were more active than the free tin compounds in terms of tin doses, with no significant difference between the number of carbons in the chain. In vivo studies in a mouse model of melanoma showed that the material functionalized with the tetraorganotin derivative significantly decreased the tumor volume.

Our research group, in collaboration with Prof. Hrstka, continued studying a multitude of silica-based systems incorporating organotin(IV) derivatives and synthesizing more specific nanodrugs. For example, mesoporous silica nanoparticles (MSN) of around 90 nm were functionalized with the polyamino ligand N^1^-(3-trimethoxysilylpropyl)diethylenetriamine to attach folic acid (FA) and, subsequently, with the “SnPh_2_” moiety through the epoxy opening reaction in a basic medium. The presence of FA helped nanoparticle accumulation in cancer cells and this induced an enhanced apoptosis and ROS generation in cancer cells [87].

Furthermore, our group, in collaboration with Fischer-Fodor’s team, developed a more specific study using functionalized MSN and MSU-2 with a triphenyltin(IV) derivative and different percentages of FA to analyze the interaction with FOLR1 receptors [88]. After an extensive characterization, it was observed that the biological action of these systems is due to their action as nonclassical drug delivery systems (the action of the entire system itself and not to the release of soluble tin-containing species), thus giving a new way of thinking about nanocarriers, where one is able to control the functionalization of both the desired metallodrug and additional molecules of biological interest. In this context, and to improve the transport processes of these materials to cells, an in vitro study was carried out using transferrin as a formulation protein and the systems were tested against the ovarian cancer cell line A2780, showing that the functionalization with transferrin protein at only 1% by mass gave the materials a higher selectivity and accumulation in the target area, showing a high cytotoxic activity of up to more than 50 times compared to the commercial drug cisplatin. In addition, the systems had capacity for modulating and downregulating the growth and transcription factors [74].

**Figure 8:**
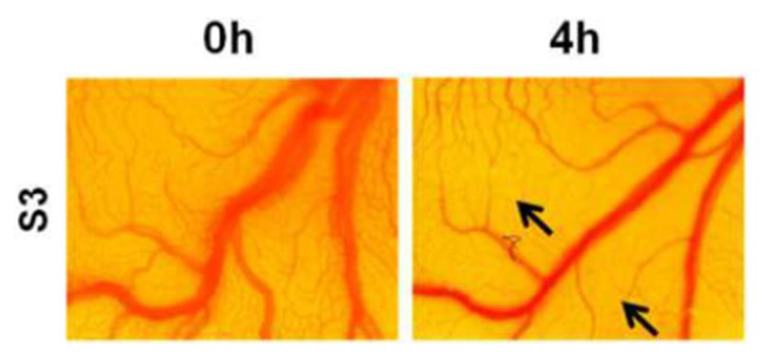
Antiangiogenic properties of MSNMP-SnPh_3_ (S3) in chick embryo assay (CAM) at 4 h post-treatment. Arrows show the main areas where reduction in the vascularization is visible [89]. Reproduced with permission. Copyright © Elsevier.

To go one step further in this field, our group, in collaboration with Prof. Patra, performed a genotoxicity study of organotin(IV) functionalized mesoporous silica nanoparticles in a chick embryo model (Figure 8), proving a high antiangiogenic capacity of the systems as well as an increase in reactive oxygen species as a plausible mechanism for the enhanced cytotoxic activity in this type of nanosystem [89].

**Figure 9:**
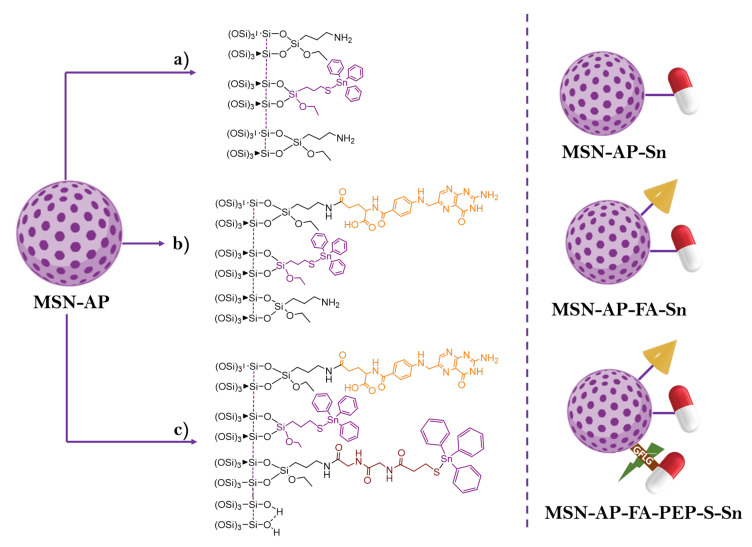
General synthetic strategy for the preparation of the three mesoporous silica systems. Purple moiety: organotin(IV) cytotoxic compound, red: enzyme-responsive linker; orange: folate fragment. (**a**) Incorporation of the cytotoxic agent, (**b**) incorporation of the targeting molecule and (**c**) incorporation of organotin(IV) fragment through an enzyme-response peptide. Adapted from reference [90]. Copyright © Authors of the publication (open access).

Of special relevance are the recent studies of our team, in collaboration with Filice’s research team, focusing on the in vivo test of organotin(IV)-functionalized silica-based materials [90,91]. In one of these studies, three different nanostructured silica systems were prepared (Figure 9), namely, a triphenyltin(IV) bound to an S-containing linker and grafted onto MSN, the same system also incorporating folic acid and a sophisticated system with folic acid and the organotin derivate incorporated either by grafting or through an enzyme-responsive peptide in order to facilitate the tin release. Furthermore, a fluorophore (Alexa Fluor 647) was incorporated to the final materials to show their potential activity in vivo against triple negative breast cancer MDA-MB-231. The materials had a good activity in studies of MTT cell viability, migration and suppression of the tumor volume. The combination of the covalently bound metallodrug and the specific release with the enzyme-responsive peptide showed that this novel nanosystem is a promising multitherapeutic material with a realistic potential to be studied in clinical trials in the future after studying some additional pharmacological parameters [90].

**Figure 10:**
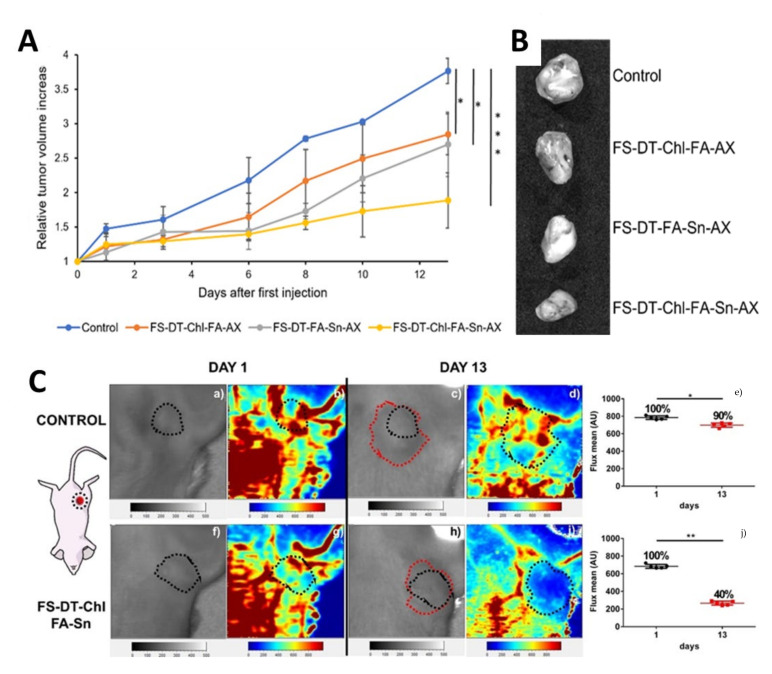
Evaluation of synergistic antitumor activity of theranostic nanoplatform in TNBC mice models. (**A**) Relative tumor volume increase during the treatment with the nanoparticles or placebo (control). (**B**) Ex vivo tumor images for all mice treated with silica nanoparticles or placebo. Significance was calculated by unpaired t-test of one-way ANOVA. *: *p* < 0.05 or significant statistical difference between the groups of data; ***: *p* < 0.001 or highly significant statistical difference between the groups of data. (**C**) Laser Doppler perfusion imaging (LDI) of tumor areas. (**a**) bright field image of control mouse, day 1 (black dotted line: tumor area at the beginning); (**b**) laser doppler imaging of control mouse, day 1; (**c**) bright field image of control mouse, day 13 (red dotted line: tumor area at the endpoint); (**d**) laser doppler imaging of control mouse, day 13; (**e**) comparison of blood perfusion in tumor area of control mouse; (**f**) bright field image of treated mouse, day 1 (black dotted line: tumor area at the beginning); (**g**) laser doppler imaging of treated mouse, day 1; (**h**) bright field image of treated mouse, day 13 (red dotted line: tumor area at the endpoint); (**i**) laser doppler imaging of treated mouse, day 13; (**j**) comparison of blood perfusion in tumor area of treated mouse. Significance was calculated by unpaired t Student test. *: *p* < 0.05 or significant statistical difference between the groups of data; **: *p* < 0.01 or highly significant statistical difference between the groups of data. [91]. Copyright © Authors of the publication (open access).

A more recent study focused on the evaluation of the therapeutic action against triple negative breast cancer of fibrous silica nanoparticles (FSP) synthesized by a hydrothermal method and postfunctionalized with a cocktail of drugs, namely, an organotin(IV) compound and the FDA-approved drug chlorambucil. The results showed a relevant in vitro cytotoxicity and antiproliferative activity (wound healing) in MDA-MB-231 cells, demonstrating that FSP materials functionalized with both drugs have a great therapeutic capacity which is also accompanied by a substantial mitigation of cell migration in a synergistic manner. The subsequent in vivo studies in a triple negative breast cancer mice model showed, thanks to the theranostic capacity of the system due to the incorporation of the Alexa Fluor agent, a selective bioaccumulation in the tumor area was observed by imaging techniques and the system promoted a substantial reduction in tumor volume after 13 days of treatment and a significant antiangiogenic effect in the tumoral area (Figure 10). In addition, the treatment with these materials seemed not to be aggressive, as all the biochemical parameters remained stable compared with the control mice [91]. In addition, the systems showed an antiangiogenic effect, decreasing the blood vascularization and the blood flux after treatment (Figure 10C).

#### 3.1.4. Other Metals in Cancer

There is a wide range of metals besides platinum, titanium and tin that have shown a high potential in the field of biomedicine and, specifically, in cancer therapy [92]. One of the most important metals is ruthenium. Concretely, Ru(III) complexes have good properties in the pharmacological field as their mechanism of action in cell death is very different from that of platinum complexes, principally due to the octahedral structure of the ruthenium center. This octahedral arrangement gives great advantages compared with platinum since it offers the possibility of two more binding sites. Ruthenium also mimics iron metabolism and transport by binding to the transferrin protein, increasing its accumulation in tumor cells [93]. The first Ru compound to enter the clinical phase was NAMI-A which demonstrated a great antimetastatic capacity regardless of the type of tumor and reached phase II in combination treatments with gemcitabine. Two more ruthenium complexes, KP1019 and BOLD-100, have also been quite promising, reaching phase I clinical trials [94]. In this context, the use of silica-based materials as nanocarriers of different ruthenium complexes has clearly increased during recent years. Citing some of the most recent reports in the field, one must mention the excellent work of Sun and co-workers, who designed a nano-Fenton reactor loaded with a commercial Ru complex and L-ascorbyl palmitate, that demonstrated a good in vitro and in vivo activity against gastric carcinoma [95]. Another interesting study was conducted by M. Mladenović et al., who designed pH-responsive hydrazone functionalized silica-based systems that were treated with a ruthenium metal complex, releasing only the metal-containing cytotoxic fragment in the tumor environment [96].

**Figure 11:**
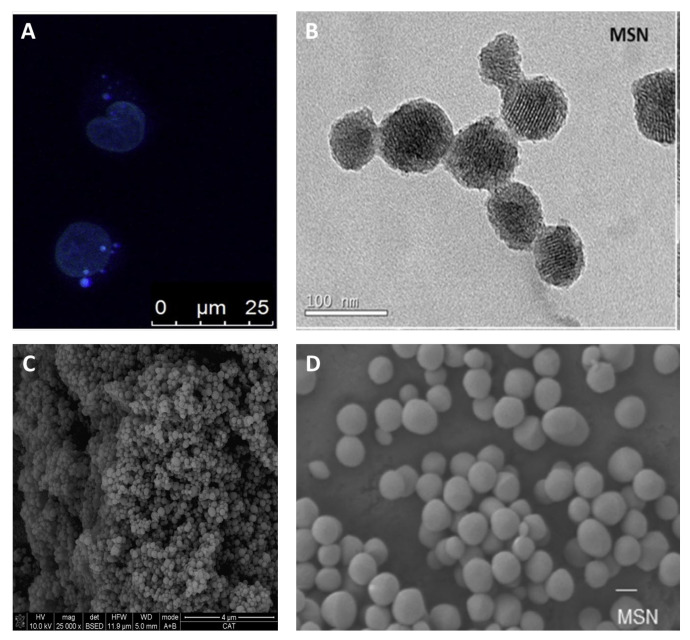
(**A**) Microscopy images of the internalization of SBA-15 in HL-60 cells, dark blue dots are due to accumulation of nanostructured material inside the cells [97], (**B**) TEM of MSN [98], (**C**) SEM of MSN [99] and (**D**) SEM of MSN, scale bar 200 nm [100]. Reproduced with permission. Copyright © Elsevier (**A**), © Royal Society of Chemistry (**B**), and © American Chemical Society (**C**,**D**).

Regarding the contribution of Spanish researchers, three reports on silica materials loaded with ruthenium complexes have been published in recent years. The work of S. Rojas et al. was based on the encapsulation of two ruthenium complexes in a silica prepared by a hydrothermal method (MCM-41) and another silica prepared by a sol-gel method (SBA-15), demonstrating an excellent activity against the leukaemia cells HL-60 and observing that the mechanism of cytotoxicity associated with the metal compounds could be caused by the interaction with the cysteine of biomolecules [97] (Figure 11). Subsequently, our group, in collaboration with Dr. Gasser’s research team, performed a comparative work on the functionalization of a ruthenium polypyridyl complex on MSN by both physisorption and two types of covalent anchoring, showing preliminary results of their promising phototherapeutic activity [98]. This was followed on with the preparation of two Ru(II) polypyridine complexes covalently bound to silica functionalized with folic acid as the targeting fragment. The systems produced high amounts of singlet oxygen upon light irradiation and, therefore, an interesting photoactivity against cancer cells [99]. In addition, Martínez-Carmona et al. published an interesting article on an octahedral ruthenium complex loaded on MSN-type nanoparticles (Figure 11), studying its anticancer capacity against glioblastoma and ovarian cancer. The work showed the different activity in the systems in terms of the pH-dependent release, cell viability, internalization and cell cycle analysis, demonstrating the importance of nanoformulation design and application conditions in therapy [100].

Another metal with high anticancer potential is gallium and it is currently making inroads among alternative metallodrugs to platinum derivatives. Gallium is interesting as it has properties and biochemical pathways similar to those of the Fe^3+^ ions. Gallium is very stable in the +3 oxidation state and is redox stable in physiologic conditions and this leads to strong interactions with proteins that usually lead to the loss of protein function and promotes cell death [101]. Despite its promising characteristics, reflected by several papers of gallium compounds against cancer [102], to the best of our knowledge there is only one report in which this metal is functionalized in silica [103]. Here, silica was functionalized with an aminopropyl ligand and loaded with gallium nitrate and curcumin to study the cytotoxic potential against MCF-7 cells. The study showed that the nanosystem interacts with oncoproteins and mitochondrial proteins, causing tumor cell death with a different mechanism than those found in the case of other metallodrug-functionalized silica-based materials.

Finally, gold systems must also be mentioned due to their ease of synthesis in different shapes, sizes, functionalities and surface plasmon resonance (optical properties) that confer particular properties to this kind of system in a biological context. However, there is only a single study concerning the incorporation of gold as organometallic complexes in silicas. Malekmohammadi and co-workers [104] prepared an aminated mesoporous silica, to which they incorporated folic acid to improve internalization by endocytosis and loaded it with a gold(III) complex. The new nanosystem demonstrated an efficiency of almost twice that of the isolated gold complex, demonstrating the advantage of using silica as a carrier for metallodrugs. On the other hand, Au nanoparticles have been the subject of many studies in cancer diagnosis and therapy [105]. Most commonly, gold is synthesized as nanometer-sized particles, so some recent studies combining silica with gold have been based on a coating of the latter in order to load the nanosystem with another cytotoxic agent to determine and optimize a controlled release and a sustained cytotoxic activity against cancer cells [106,107].

**Figure 12:**
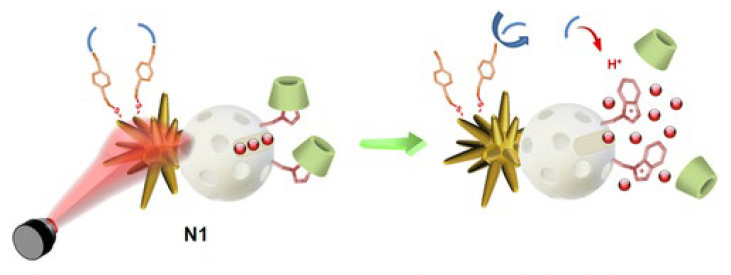
Janus gold nanostar–mesoporous silica nanoparticle (white sphere) for the release of doxorubicin (red dots). Cyclodextrin gates are in green colour. Reproduced with permission from [108]. Reproduced with permission. Copyright © American Chemical Society.

In this context, there are contributions from outstanding Spanish researchers such as the work of the Martínez-Máñez group [108,109] which synthesized silica- and paraffin-coated gold nanostars for the controlled release of doxorubicin when the nanosystem was subjected to a near-infrared laser beam due to paraffin melting [108]. In addition, they prepared Janus-type silica NPs loaded with the doxorubicin, where one side was functionalized with gold nanostars and the other with cyclodextrin (Figure 12). Upon incident near-infrared radiation, succinic acid was generated, opening the “gates” that released the doxorubicin into the tumor cells [109].

### 3.2. Antibacterial Activity

Presently, deaths caused by drug-resistant diseases are more than 700,000 per year. The WHO estimates that, in 2050, the antimicrobial resistance (AMR) will be an alarming threat to global health as it is expected to be one of the major causes of death, even above cancer [110]. In 2019, WHO General Director Tedros Adhanom Ghebreyesus said that superbugs are “*one of the most urgent health threats of our time*”.

The term “superbugs” is used for those bacterial strains that have developed resistance to most of the known antibiotics. This is a natural process by which bacteria or fungi adapt to the drugs that are administered to stop them or kill them, thus making the drug less and less effective to such an extent that it has no impact on them. AMR not only directly affects humans, but also animals and plants, having the potential to affect them at any stage of their life cycle. Penicillin, discovered by Alexander Fleming in 1928, was the first antibiotic drug to be commercialized. However, since its industrial production in 1941, it only took a year for the first resistant germen, a strain of *Staphylococcus aureus*, to appear, because of this problem, it is necessary to encourage the innovation for new strategies and drugs for preventing or breaking the antibiotic resistance in order to reduce the spread of infections and avoid a serious crisis, both at an economic and global health level.

There are many classes of antibiotics depending on the target bacterial strain and the necessary mechanism of action. A general classification is based on the potential for inhibiting bacterial growth (bacteriostatic drugs) or if they are able to kill the bacteria (bactericidal drugs). The use of new antimicrobial agents is important to combat drug resistance by designing new drugs or systems with a high toxicity and bacterial inhibition.

Although there are a wide variety of metal-based systems [111] (especially based on copper [112] and silver complexes [113]) that have been used in antibacterial studies, this part of the present review will focus on the use of silica-based materials as the support of metal complexes or other metal species as new therapeutic agents against bacteria. There is a very recent review covering the use of MSN-based systems in this field [114], however, covering only the ways of functionalization of MSN but not the use of different metallodrugs.

#### 3.2.1. Silver

The use of silver for antibacterial purposes has been so for thousands of years. Silver, and in particular silver nanoparticles (AgNPs), have multiple biological properties such as antibacterial, antiviral and antifungal capacity. The modulation of the nanometer size and morphology of the NPs allows a strong interaction with the biomolecules of the cell wall, weakening the physical separation between the bacteria and the medium and being able to infiltrate inside the microbe. The specific mechanism of action is complex, although it seems that silver ions act on bacterial membranes, degrading them, while on the other hand, they interact with enzymes and DNA, preventing their proliferation. In addition, AgNPs have an antibiofilm activity, which prevents bacteria or other pathogens from adhering to and colonizing surfaces in an almost irreversible manner [115].

Silica-silver formulations compensate for the disadvantages produced by the use of isolated silver nanoparticles or silver complexes. A very practical way to synthesize this kind of hybrid system is the preparation of core–shell systems [116] or the encapsulation of AgNPs in silica pores prepared with a nonsurfactant templating method [117]. Ag-SiO_2_ hybrid materials allow greater control of the silver release, improving its selectivity and antibacterial activity which can act against a great variety of bacterial strains.

In this field of work, some Spanish groups have contributed to the development of silver-functionalized silica-based materials as antibacterial systems. For example, a collaborative study between groups from Brazil, Argentina and Spain designed a composite based on silica-polyethylene by polymerization reactions which allowed a covalent functionalization with AgNPs at different pH. These systems were studied against three bacterial strains, one gram-positive (*S. aureus*) and two gram-negative (*Salmonella spp.* and *E. coli*, and showed that the antibacterial capacity depended on the synthetic route with the size of the nanoparticles being responsible for the inhibition of bacterial growth through surface contact, without the need for silver leaching [118]. In another study, the groups of González and Luque-García designed an MCM-41 with silver bromide NPs and Ag@SiO_2_ core–shell systems as efficient nanocarriers against *Mycobacterium tuberculosis*. Both systems demonstrated a high antibacterial activity presumably due to the bacterial cell membrane deterioration being more accentuated for those formed by silver bromide NPs, since they have a greater capacity of penetration in the membrane due to the release of Ag^+^ ions [119].

**Figure 13:**
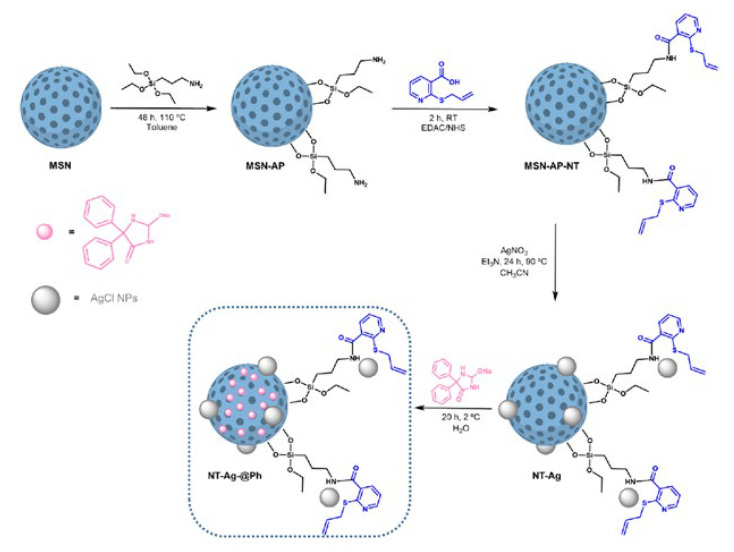
Synthesis scheme of mesoporous silica nanoparticles (blue sphere) loaded with a dual system of a nicotinic acid (pink spheres) and silver chloride NPs (grey spheres) [120]. Reproduced with permission. Copyright © Authors of the publication (open access).

In addition, recent work of our research team, in collaboration with Dr. Aguilera-Correa and Dr. Páez, focused on the synthesis and characterization of a hybrid system of mesoporous silica decorated with a nicotinic acid and silver chloride NPs (Figure 13). The system showed a promising antibacterial activity against three different planktonic *P. aeruginosa* strains. These strains are known to have a great facility for generating biofilms; however, the treatment of this silica system obtained very good results in terms of minimal biofilm inhibitory concentration (MBIC) and minimal biofilm eradication concentration (MBEC) values compared to silver NPs alone [120].

Regarding systems based on silica and silver complexes, to the best of our knowledge, to date there is only a single paper by Y. Kuthati et al. that reported the preparation of IBN-4 type silica by hydrothermal treatment, loaded with a silver complex via the reaction with a pH-sensitive hydrazone ligand. The nanosystem was able to inhibit the biofilm formation of *E. coli*, *B. subtilis*, *S. aureus* and *S. epidermis*, greatly increase the production of ROS and induced protein leakage and DNA fragmentation, and, thus, bacterial death [121].

#### 3.2.2. Copper

As in the case of silver, copper has been used as a treatment for infections for thousands of years since the days of the Egyptians and Romans. Copper is essential in most of the organisms for important processes such as cellular respiration or photosynthesis. Specifically, bacterial cells need this metal for metabolizing the enzyme cytochrome c oxidase, and in gram-negative bacteria, for example, the use of copper is essential to metabolize methane to methanol [122]. Interestingly, a deregulation of copper is capable of causing toxicity at the bacterial level, being able to take advantage of this characteristic for antibacterial purposes. In fact, copper is the first, and the only metal, with antibacterial properties approved in 2008 by the Environmental Protection Agency (EPA). Therefore, it has highly promising properties and applicability at a very low cost. A great advantage of copper is that it does not induce resistance in bacteria. Copper is present in nature and, until now, no microorganisms have shown resistance to it. Copper also has a multitherapeutic way of acting, allowing different mechanisms of antibacterial activity which go down different routes to other antibiotics. This is the main reason for copper compounds and materials hindering the development of resistance and killing microorganisms before they can replicate, precluding the development of resistance which can be transmitted to the next generations of microbes.

The molecular and chemical mechanism on how copper acts against bacteria is still not known with accuracy, although several hypotheses have been postulated in several scientific studies [123]. Firstly, it seems that copper ions are capable of breaking down proteins from the bacterial wall and intervening in the synthesis, allowing the leakage of bacterial nutrients and other cellular content, which in a final step leads to the death of the bacteria [124]. On the other hand, it seems that copper also interacts with the transport, storage or other kind of proteins displacing other metal ions from their binding site. This can also happen with bacterial DNA and causes a degradation of DNA or dysfunction of the ion tasks in the bacteria, leading the microorganism to cell death.

**Figure 14:**
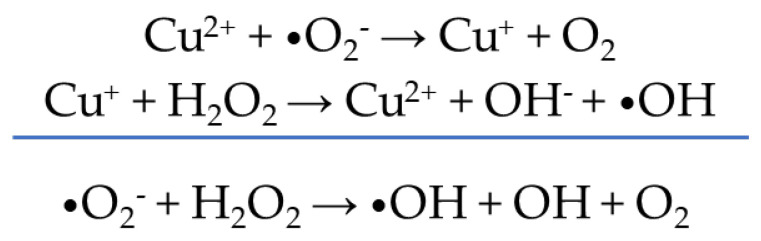
Mechanism of formation of ROS from Cu^2+^.

Finally, it has also been observed that copper ions can generate, by the interaction of electron carriers and reactive oxygen species (ROS), through the reaction known as Fenton/Haber–Weiss, causing oxidative stress to the cell (Figure 14). However, this approach is still not entirely clear since the use of copper in some bacteria under anaerobic conditions produces greater cellular damage than in the presence of oxygen, which shows that the action of copper does not follow a single mechanism [125].

There are many studies in the literature regarding using copper in different coordination environments; for example, copper oxides, copper(II) Schiff-base complexes and copper nanoparticles [111]. However, the investigation dealing with the incorporation of copper compounds (not copper nanoparticles) in silica materials is very limited.

In 2015, H. Palza and co-workers used a silica nanoparticle to interact with CuCl_2_ and to form nanocrystals of paratacamite (Cu_2_(OH)_3_Cl). This system was capable of releasing copper in an aqueous medium, producing an antibacterial activity [126]. Subsequently, W. Zhang et al. synthesized a Schiff-base copper(II) complex that was supported in silica NPs through an aminopropyl ligand in order to make a bactericide less polluting and harmful to plants [127]. The study showed that, with this new design, a lower amount of copper was needed to obtain a good bactericidal activity, compared with the free copper complex. In an analogous work, L. Tahmasbi et al. designed a system based on MCM-41 incorporating a Cu(II) and a Ni(II) Schiff-base complex. These metal-containing silica systems were also acting as carriers of gentamicin with very interesting properties [128].

A very different strategy involving copper as a synthetic template was carried out by the group of Halbus. This study showed that the use of copper NPs as a template for porous silica nanoparticles, and a subsequent functionalization with a glycil ligand and boronic acid, leads to new antibacterial systems thanks to the interactions with bacterial membranes [129].

The main contribution of Spanish researchers to this field has been carried out by our group and those of Montalvo and Martín. We have recently published two reports in which copper complexes have been anchored to mesoporous silicas with antibacterial capacity [130,131]. The first study was focused on the preparation of MSN silicas (by sol-gel methods) functionalized with a maleamate ligand capable of coordinating copper(II). This system showed a great inhibitory and bactericidal capacity against different strains, with a similar or even higher activity than commercial antibiotics. The antibacterial activity in the Cu-functionalized mesoporous silica systems associated their antibacterial activity to their catalytic potential in selective oxidation processes to form ROS [130]. In the second study, we described the preparation by a sol-gel method and characterization of nanostructured silica SBA-15 functionalized with an imidazole and a maleamic ligand for the subsequent incorporation of coordinated copper ions. These systems showed a good antimicrobial activity against a gram-positive (*S. aureus*) and a gram-negative strain (*E. coli*), observing also, through electrochemical studies, that their mode of action is presumably due to an interaction with amino acids involved in the synthesis of the peptidoglycan of the bacterial wall [131].

The other Spanish contribution in this field was focused on the design of a composite silica foam with a copper salt (using microwaves) and copper nanoparticles (by green chemistry reduction). These systems showed an outstanding antibacterial activity against *E. coli* and *S. aureus* without the need of a metal ion release [132].

Despite the advancement in nanomedicine, there is still a lot to undertake in this field to promote the use of immobilized silver or copper complexes on silica nanoparticles, promising new structures and systems are yet to be explored and would undoubtedly help in the design of effective antibacterial systems against superbugs.

## 4. Conclusions

This review gives an overview of the current advances in systems based on silica nanostructured materials as carriers of drugs and molecules of biological interest. In particular, the use of silica systems as carriers for metallodrugs, although still far from being implemented in clinical trials, is an innovative alternative to the use of free metal complexes in therapies against cancer and bacterial infections since they improve problems with dosage by requiring a much lower amount of metal and, therefore, offering more sustainability.

Even though there is still a lot of research work to do in this field for obtaining the exact therapeutic mechanism of action of these systems to promote their use in humans, the studies carried out during the last 15 years have given some insights on the mechanism of therapeutic action of free metallodrugs, reaching several conclusions agreed by the scientific community to interpret the action pathways of the compounds. However, when metallodrugs are supported on silica, as their therapeutic activity can be modulated depending on the design and loading of the systems to generate more reactive oxygen species, changes in growth factors or interaction with different proteins, there are several steps that need to be clarified for going further to clinical applications.

Nevertheless, these systems have a high versatility, as the classic systems of the transport of metallodrugs, initiated by the pioneering work of the Spanish group of Vallet-Regí, have been based on the absorption of the drugs in porous silicas, followed by a controlled release in the target area through “intelligent gates”, changes in pH or specific interactions, where the metallic load accumulates thanks to the EPR effect, while the design of nonclassical release systems based on silica have become, in recent years, a great competitor for the classical model and an interesting alternative.

Thus, the nonclassical release systems have shown a great activity against a multitude of cancer cell lines in vitro, and some cancer mice models in vivo. In addition, nonclassical metallodrug-functionalized silica nanostructured systems have also been used against different bacterial strains. Interestingly, in this field, several Spanish researchers are key contributors in preparing nonclassical metallodrug-functionalized drug delivery systems that do not require the release of the metal complexes for their therapeutic activity to take place.

For an adequate and efficient design, synthesis and study of the biological properties of metallodrug-functionalized silica-based nanomaterials, a thorough characterization of each material and each synthetic step associated with the functionalization is necessary, because every little change in the system can cause significant changes in the therapeutic biological properties of the system, which is crucial for its final use.

Finally, it is important to note that the versatility in the design and functionalization of silica allows not only the possibility of improving existing therapies in cancer and bacterial conditions, but also to expand the use of these systems to other therapeutic approaches such as neurodegenerative diseases treatment [133], bone regeneration [134] and surgical operations due to their antibacterial properties [135].

This review, therefore, has aimed to open the field of vision of researchers to expand the knowledge of these nanosystems, the valuable contribution of Spanish researchers in this field and to show the perspective to exploit the potential application of these systems in the future.

## Figures and Tables

**Table 1:** Principal metals used in anticancer and antibacterial applications highlighting references with significant contribution of Spanish authors.

Metal	Application	Reference
Pt	Anticancer (phototherapy)	[19]
Sn	Anticancer (pancreatic carcinoma, erythroleukemia, glioblastoma)	[20]
Ti	Anticancer (Adenocarcinoma and cervix cancer)	[21]
Ru	Anticancer (prostate, breast cancer, cervix and liver cancer)	[22]
Ga	Anticancer (anaplastic thyroid cancer, head and neck tumor, lung carcinoma, ovarian cancer, colon carcinoma)	[23]
Au	Anticancer (colon carcinoma)	[24]
Ag	Antimicrobial (*S. aureus*, *E. coli*, *P. aeruginosa* and Mycobacteria strains *M. bovis* and *M. tuberculosis*) and anticancer (breast cancer and hepatocellular carcinoma)	[25]
Cu	Antibacterial (*S. aureus*, *E. faecalis*, *E. coli* and *P. aeruginosa*), antifungal (*C. albicans*, *C. glabrata*, *A. fumigatus* and *A. alternata*) and anticancer (breast and colorectal cancer)	[26]

## Data Availability

Not applicable.
